# Simultaneous Quantitative Susceptibility Mapping of Articular Cartilage and Cortical Bone of Human Knee Joint Using Ultrashort Echo Time Sequences

**DOI:** 10.3389/fendo.2022.844351

**Published:** 2022-02-22

**Authors:** Ming Zhang, Zhihui Li, Hanqi Wang, Tongtong Chen, Yong Lu, Fuhua Yan, Yuyao Zhang, Hongjiang Wei

**Affiliations:** ^1^ School of Biomedical Engineering, Shanghai Jiao Tong University, Shanghai, China; ^2^ Department of Radiology, Ruijin Hospital, School of Medicine, Shanghai Jiao Tong University, Shanghai, China; ^3^ School of Information and Science and Technology, ShanghaiTech University, Shanghai, China

**Keywords:** quantitative susceptibility mapping, ultrashort echo time, articular cartilage, cortical bone, computed tomography

## Abstract

**Background:**

It is of great clinical importance to assess the microstructure of the articular cartilage and cortical bone of the human knee joint. While quantitative susceptibility mapping (QSM) is a promising tool for investigating the knee joint, however, previous QSM studies using conventional gradient recalled echo sequences or ultrashort echo time (UTE) sequences only focused on mapping the magnetic susceptibility of the articular cartilage or cortical bone, respectively. Simultaneously mapping the underlying susceptibilities of the articular cartilage and cortical bone of human *in vivo* has not been explored and reported.

**Method:**

Three-dimensional multi-echo radial UTE sequences with the shortest TE of 0.07 msec and computed tomography (CT) were performed on the bilateral knee joints of five healthy volunteers for this prospective study. UTE-QSM was reconstructed from the local field map after water-fat separation and background field removal. Spearman’s correlation analysis was used to explore the relationship between the magnetic susceptibility and CT values in 158 representative regions of interest of cortical bone.

**Result:**

The susceptibility properties of the articular cartilage and cortical bone were successfully quantified by UTE-QSM. The laminar structure of articular cartilage was characterized by the difference of susceptibility value in each layer. Susceptibility was mostly diamagnetic in cortical bone. A significant negative correlation (r=−0.43, p<0.001) between the susceptibility value and CT value in cortical bone was observed.

**Conclusion:**

UTE-QSM enables simultaneous susceptibility mapping of the articular cartilage and cortical bone of human *in vivo*. Good association between susceptibility and CT values in cortical bone suggests the potential of UTE-QSM for bone mapping for further clinical application.

## Introduction

Cartilage and bone, providing stiffness and strength, are two fundamental knee joint structures. Articular cartilage degradation is regarded as a typical feature involved in the development of osteoarthritis (OA) (1). Bone tissues consist of two types, cortical bone and cancellous bone. Cortical bone plays a key role in the mechanical competence of bone, which occupies approximately 80% of the total bone mass (2) and accounts for 80% of all sites where bone fractures occur (3, 4). Therefore, a useful tool to noninvasively investigate the microstructure of the articular cartilage and cortical bone is of great importance for clinical evaluation.

Computed tomography (CT) has been widely used in clinical practice for bone mapping. However, CT involves ionizing radiation and exhibits poor contrast for soft tissue structures, e.g., the cartilage. Magnetic resonance imaging (MRI) can overcome the disadvantages of the X-ray-based technique and offers superior image contrast in soft tissues. However, there are signal voids in regions (e.g., the cortical bone) (5) with rapid signal decay (0.1~1 msec) (6) on MRI images using conventional sequences with echo time (TE) of a few msec or longer. The MRI-based ultrashort echo time (UTE) sequences, which collect signals immediately after pulse excitation resulting in a very short TE (typically less than 0.1 msec), provide the feasibility for direct cortical bone imaging (7–9).

Quantitative susceptibility mapping (QSM) is a post-processing MRI technique that quantitatively estimates the underlying magnetic susceptibilities of tissues, typically using gradient recalled echo (GRE) sequences (10, 11). Since magnetic susceptibility is an intrinsic physical property of tissue, this unique contrast mechanism has made QSM a promising tool for tissue quantification and disease detection, such as for early brain development (12), brain aging (13, 14) and diseased brain (15, 16). Recently, researchers have applied QSM in the musculoskeletal system (17–20). However, studies using GRE sequences (GRE-QSM) failed in mapping the susceptibility of cortical bone due to signal void and low signal-to-noise ratio (SNR) at a typical TE longer than one msec. Therefore, the cortical bone regions were usually masked out for QSM processing due to unreliable susceptibility values measured from the local field map (17). Luckily, benefiting from the ability of the UTE sequence for detecting rapidly decayed signals, UTE-based QSM (UTE-QSM) could be capable of quantifying the susceptibility property of cortical bone. However, related studies mainly focused on imaging the cortical bone and didn’t quantify soft tissue susceptibility. UTE-QSM has not been applied to explore the articular cartilage of human *in vivo* (21, 22). Simultaneous susceptibility mapping of the articular cartilage and cortical bone with a single MRI scan is still lacking and highly needed.

In this study, we aimed to simultaneously quantify the magnetic susceptibilities of the articular cartilage and cortical bone of the human knee joint using UTE-QSM. To explore the clinical potentials of UTE-QSM, the relationship between the susceptibility and CT values in cortical bone was also assessed.

## Materials and Methods

### Subjects

This study was approved by the Human Ethics Committee of Shanghai Jiao Tong University. Five healthy young volunteers (4 males, mean age of 23.6 years old) were included in this study with both MRI and low dose CT examinations on bilateral knee joints. No history of knee injury from the participants was reported. All participants were informed of the experiment protocol and informed consent was provided from each subject. The time interval between MRI and CT examinations was within a month.

### CT Examination

The low dose CT scan (volume CT dose: 6.74 mGy, dose length product: 132.8 mGy*cm) for each participant was performed using a clinical CT scanner (SOMATOM Definition AS, Siemens Healthcare, Erlangen, Germany). The scanning parameters were: X-ray tube voltage of 120 kV, tube current of 100 mA; slice thickness of 0.7 mm, 252 slices; matrix size of 512×512, spatial resolution of 0.85×0.85 mm^2^.

### MRI Examination

Each subject has their right and left knee joints scanned on a 3 Tesla MRI scanner (uMR 790, United Image Healthcare, Shanghai, China). For the three-dimensional UTE sequence, a non-selective hard pulse was used to excite the whole knee joint. The free induced decay (FID) signal (i.e., ultrashort TE signal) and gradient recalled echoes (i.e., normal TE signals) were collected in the center-out and center-in radial trajectories, respectively (23). Two continuous UTE sessions, each with one ultrashort TE and two normal TEs, were taken for imaging each limb of the participants without repositioning, resulting in 6 TEs in total. TEs of the first UTE scan, 0.07/2.24/3.55 msec; TEs of the second UTE scan, 0.1/2.8/4.6 msec. Other parameters for UTE were: axial view; repetition time, 10 msec; flip angle, 8°; field of view (FOV), 180×160×144 mm^3^; image matrix, 208×184×160; spatial resolution, 0.9×0.9×0.9 mm^3^; spoke number, 40960; scan time, 7 min 6 sec per scan.

### UTE Processing

The UTE raw data from the MRI scanner was sent to a high-performance workstation for processing. The density function was calculated to compensate for the measured signals due to the nonuniform radial sampling pattern (24). Nonuniform fast Fourier transform was applied for gridding to interpolate the data from radial spokes into Cartesian grids (25). Each channel of the UTE image was combined to generate four-dimensional complex-valued data. Subsequently, image registration was performed on the magnitude images to reduce the possible error induced by slight inter-scan motion during two separate UTE scans. The FMRIB’s Linear Image Registration Tool (26, 27) with 6 degrees of freedom and a maximum rotation angle of 10° was used for registration. The transformation matrices were automatically saved and applied to the real and imaginary parts of UTE data to bring two separate UTE complex-valued images into an identical space.

### UTE-QSM Reconstruction

The water-fat separation method was applied to UTE images to eliminate the chemical shift effect due to the presence of fat in the knee joint, which could further hamper the susceptibility quantification due to the inaccurate field map estimation (28). The B_0_ field map was derived during this procedure using the graph cut-based water-fat separation algorithm (29) with the conventional six fat peaks model on a slice-by-slice basis.

A binary mask was generated by thresholding the sum-of-squares of the UTE magnitude image (15% of the maximum intensity) to exclude the background. The V-SHARP (variable-kernel sophisticated harmonic artifact reduction for phase data) algorithm (30) was used to recover the local field map from the total field map. The susceptibility map was quantitatively reconstructed using STAR-QSM (streaking artifact reduction for QSM) (31).

To compare with UTE-QSM, QSM from normal TEs (normal TE-QSM) was also calculated without the use of 2 ultrashort TEs following the same pipeline for QSM reconstruction.

The magnetic susceptibility value was not referenced to any areas in this study. The magnetic susceptibility value was reported in parts per million (ppm).

### CT Processing

For the CT image, the left and right knee joints were separated by dividing the whole CT image into two parts for following usages. The CT value was reported in Hounsfield units (HU). The background signal of the CT image was set to zero.

### Regions of Interest (ROIs) of Cortical Bone

The multi-modal registration between UTE and CT images was achieved using Advanced Normalization Tools (32) with the rigid plus affine transformation, plus symmetric image normalization manner. The sum-of-squares of the UTE magnitude image served as the reference.

The ROIs of cortical bone were extracted from the UTE magnitude images on 12~26 consecutive slices of each limb using threshold binarization since there was a relatively low signal intensity in cortical bone. The ROIs were placed both on UTE magnitude images and corresponding aligned CT images to visually check the isolation accuracy using the open-source ITK-SNAP software (33). A total of 158 ROIs were obtained. The mean magnetic susceptibility and CT values were calculated for each ROI. Detailed values could be found in the **Supplementary Materials**.

### Statistical Analysis

Spearman’s correlation coefficient (r) was used to investigate the relationship between the mean susceptibility value and CT value in cortical bone using data from 158 ROIs. The threshold of p-value for statistical significance was set as 0.05. The correlation analysis was conducted using MATLAB (version 2019b, MathWorks, Natick, MA) with the “*corr*” function.

## Results

**Figure 1:**
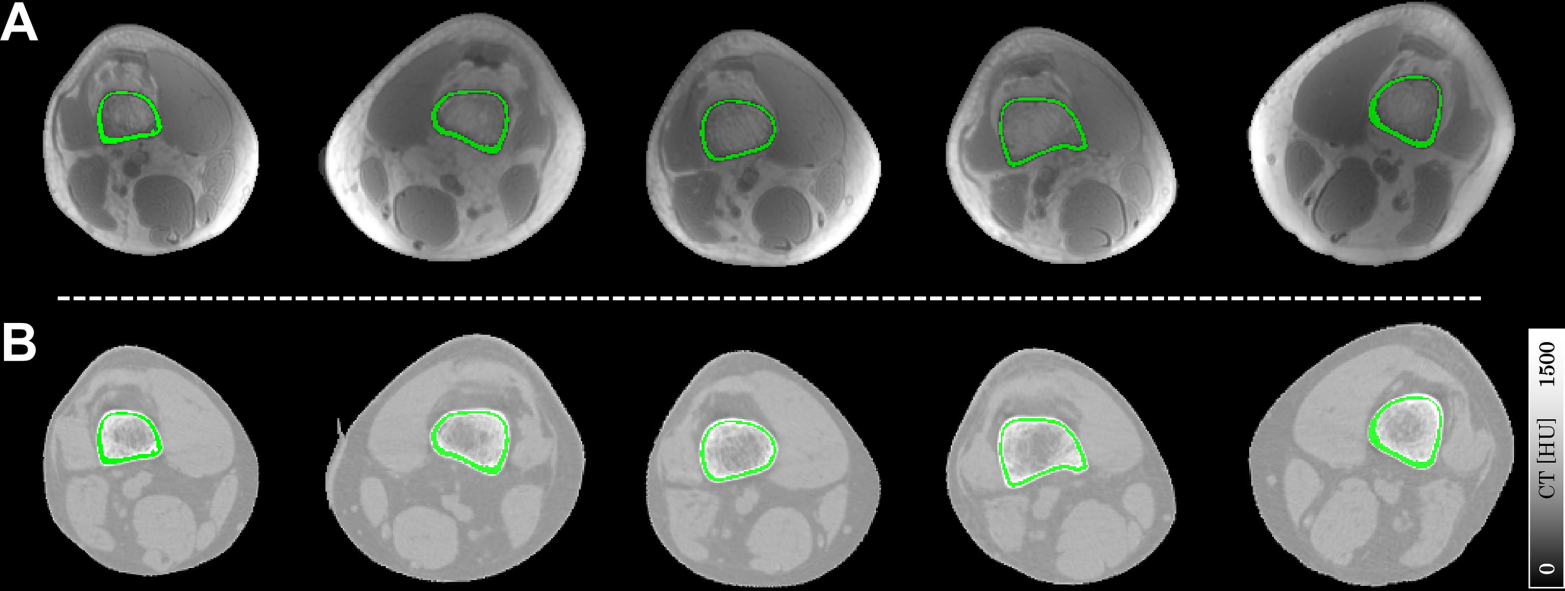
Representative slices of ROIs of cortical bone (in green) overlaid on the UTE magnitude images **(A)** and registered CT images **(B)**. Each column represents a typical ROI from an individual subject in this study.

**Figure 1** illustrates the segmented ROIs of cortical bone overlaid on their corresponding UTE magnitude images and warped CT images. The ROIs could finely match the anatomical structures of cortical bone, both on UTE and CT images.

**Figure 2:**
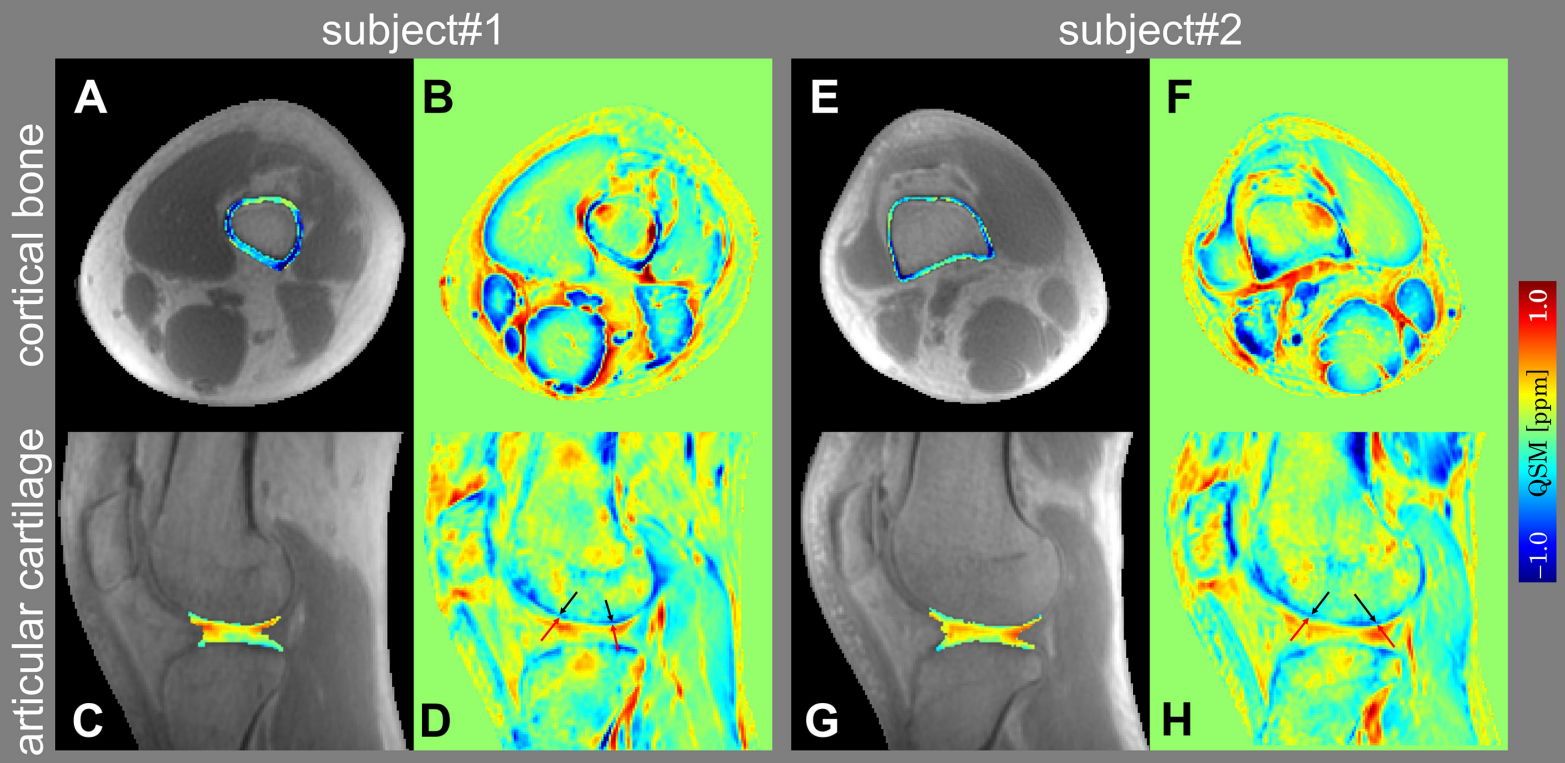
Representative slices of UTE-QSM in cortical bone **(A, E)** and articular cartilage **(C, G)** overlaid on the UTE magnitude images in axial and sagittal views of two subjects. **(B, F)** and **(D, H)** are the color-coded magnetic susceptibility maps within the whole FOV corresponding to **(A, E)** and **(C, G)**. The black and red arrows in **(D, H)** point to the subchondral bone and deep layer of articular cartilage, respectively.

**Figure 2** shows the representative slices of UTE-QSM in articular cartilage and cortical bone in the sagittal and axial views of two subjects. Gradual magnetic susceptibility change from diamagnetic to paramagnetic was visible from the deep layer (near the subchondral bone) to the superficial layer of the articular cartilage. There was a clear susceptibility difference between the deep layer of articular cartilage (light blue, less diamagnetic) and the subchondral bone (dark blue, more diamagnetic), although the deep layer of articular cartilage was relatively thin (1~2 pixels) on UTE-QSM. UTE-QSM was generally diamagnetic in cortical bone.

**Figure 3:**
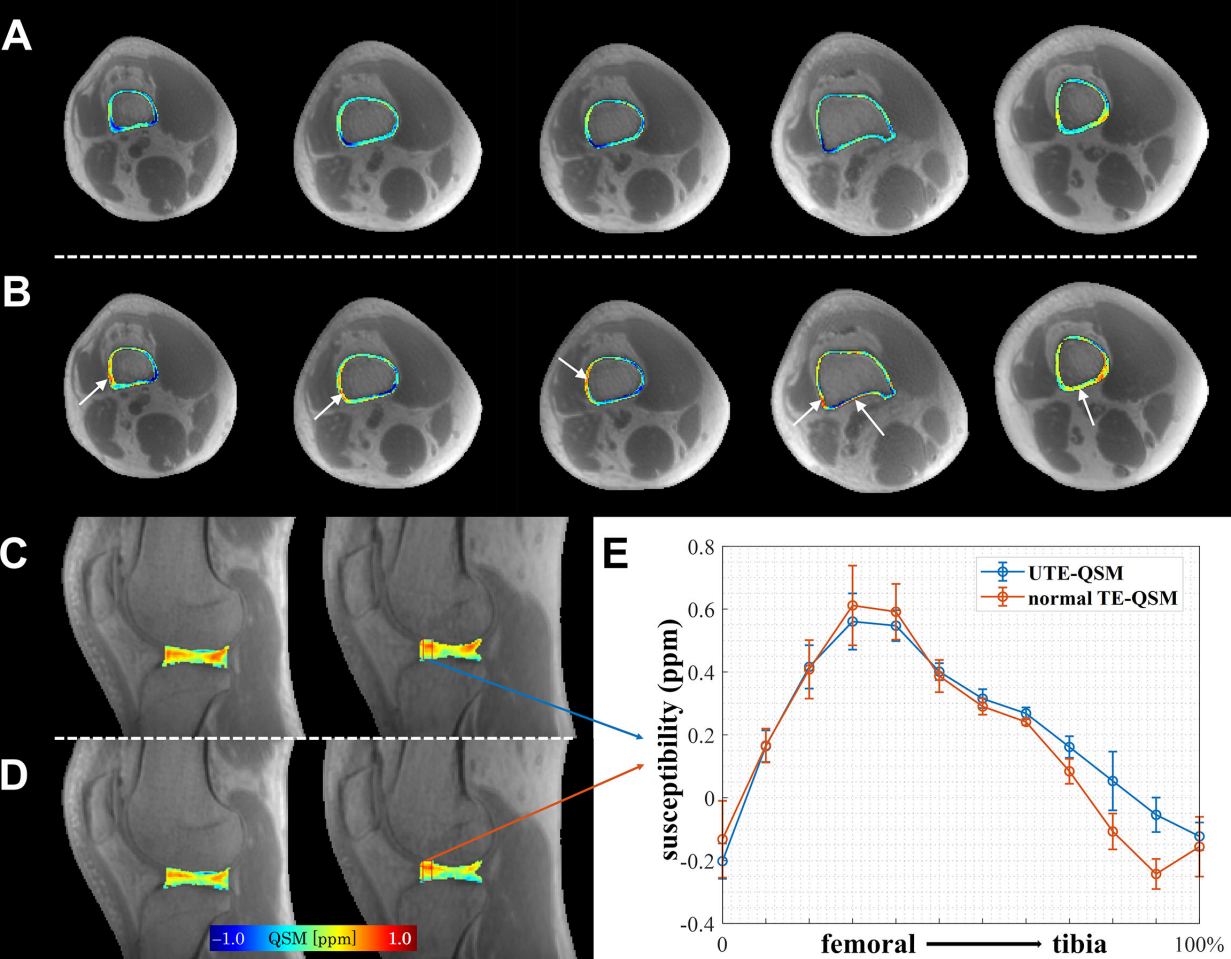
Comparison of UTE-QSM and normal TE-QSM in cortical bone and articular cartilage. In cortical bone, UTE-QSM **(A)** was more homogenously diamagnetic than normal TE-QSM **(B)** (regions indicated by white arrows). In articular cartilage, the susceptibility contrasts between UTE-QSM **(C)** and normal TE-QSM **(D)** were similar. **(E)** shows the susceptibility profiles of UTE-QSM and normal TE-QSM from femoral to tibia cartilages in the selected red box (5×12 pixels) on one healthy volunteer. Data are presented as mean±standard deviation. Each column of cortical bone in **(A, B)** or articular cartilage in **(C, D)** represents a typical image slice from an individual subject in this study.

**Figure 3** compares the reconstructed susceptibility maps from UTE-QSM and normal TE-QSM in cortical bone and articular cartilage. As could be seen, UTE-QSM exhibited an overall more homogeneous diamagnetic susceptibility in cortical bone. In contrast, certain regions exhibit paramagnetic susceptibilities in cortical bone on normal TE-QSM, resulting from inaccurate bone susceptibility quantification due to the uncertain phase measurement using relatively long TE. In articular cartilage, the susceptibility contrasts between UTE-QSM and normal TE-QSM were similar, based on the typical image slices and quantitative susceptibility profiles.

**Figure 4:**
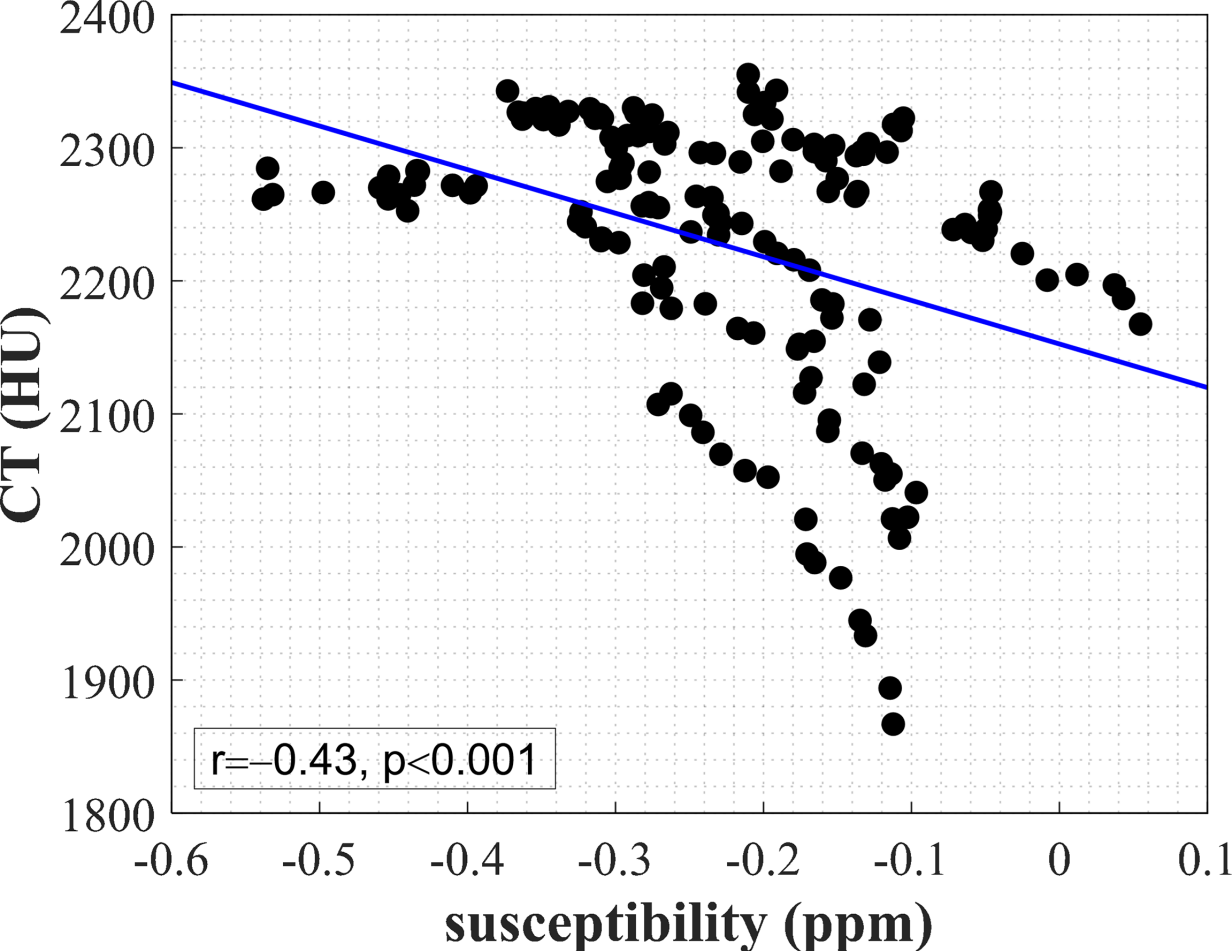
A significant negative correlation (r=−0.43, p<0.001) between the mean susceptibility value and mean CT value in 158 ROIs of cortical bone from the bilateral knee joints of 5 subjects. The blue line was obtained from linear regression.

**Figure 4** shows the result of correlation analysis of UTE-QSM versus CT values in cortical bone. A significant negative correlation (r=−0.43, p<0.001) between the mean susceptibility and CT values in cortical bone was observed.

## Discussion

In this study, we described the feasibility of UTE-QSM for simultaneous susceptibility mapping of the articular cartilage and cortical bone of the human knee joint *in vivo*. The laminar structure of articular cartilage was revealed on UTE-QSM, similar to conventional GRE-based QSM with long TE. Meanwhile, the susceptibility map in cortical bone could be accurately estimated from the collected phase data by UTE-QSM. The correlation study further demonstrated the potential of UTE-QSM to assess the cortical bone without radiation from CT.

We achieved simultaneous susceptibility mapping of the articular cartilage and cortical bone based on UTE-QSM. In previous studies using the GRE sequences, the bone regions were usually masked out to eliminate the unreliable phase measurement with low SNR. With the help of the UTE sequence, it was possible to use a larger FOV to include the whole knee joint into QSM reconstruction. The necessity of UTE sequence for cortical bone susceptibility mapping was evaluated from the comparison between UTE-QSM and normal TE-QSM. In previous studies, a high in-plane resolution with relatively thick slice thickness was often adopted (17, 21). The spatial resolution of UTE was set as 0.9 mm isotropic in this research because the cortical bone and articular cartilage were usually viewed in two different planes, the axial and the sagittal. Therefore, an isotropic spatial resolution was more preferred for simultaneous visualization.

The articular cartilage contains complex organizations. Generally, the articular cartilage can be divided into three layers, the deep, middle and superficial layers, with parallel, random and perpendicular collagen fiber orientations with respect to the subchondral bone (34). The varied collagen fiber arrangement in each layer was considered as the main susceptibility source on QSM images (17, 35). Wei et al. demonstrated the ability of GRE-QSM to reflect the collagen fiber organization in each layer of articular cartilage since the susceptibility of collagen fiber was orientation-dependent (17). A quantitative study demonstrated that the variation of magnetic susceptibility was associated with the stage of OA (36). Recent work further validated the feasibility of GRE-QSM for detecting the early microstructural change in degenerated cartilage by performing GRE-QSM on marathon runners after repetitive running (37). However, the susceptibility property using UTE-QSM in articular cartilage was not fully explored (38). We demonstrated that UTE-QSM also exhibited similar susceptibility differences at different depths of articular cartilage compared with GRE-QSM. This was mainly because that the susceptibility was expected to be TE-independent with adequate phase accumulation and SNR. However, slight differences were observed since there were intermediate steps for QSM reconstruction and possible errors could exist during these procedures due to imperfect mathematical or physical modeling (39). In this study, the deep layer of articular cartilage was relatively thinner on UTE-QSM compared with previous GRE-QSM studies, due to the limited image resolution.

In cortical bone, calcium is the primary biological source of magnetic susceptibility (40). Therefore, the susceptibility of cortical bone should be mostly diamagnetic, which was in agreement with our UTE-QSM result. The potential clinical application of UTE-QSM has been shown in several papers. For example, UTE-QSM was used for detecting hemosiderin deposition in patients with hemophilic arthropathy (22). The susceptibility was presented as high intensity in affected regions with high iron concentrations. Jerban et al. found a significant correlation between the UTE-QSM value and bone mineral density (BMD) measured by micro-CT on human cortical bone specimens, indicating that UTE-QSM could serve as an effective approach for BMD assessment (21). Although UTE-QSM could be a more general tool for knee joint mapping than GRE-QSM, more studies were still needed to fully explore the clinical value of UTE-QSM in the skeletal system, including but not limited to the knee joint (41, 42).

We found a significant correlation between the mean susceptibility and CT values in cortical bone. UTE-QSM may be considered as a potential substitution in imaging the cortical bone. There were a few studies that have attempted to explore the relationship between QSM and CT values. Dimov et al. found that UTE-QSM negatively correlated with CT value in diamagnetic susceptibility regions, such as in the trabecular and cortical areas on a porcine specimen (38). Oshima et al. conducted comprehensive experiments for assessing the general association between susceptibility and CT values with both phantom and human studies using GRE-QSM (43). The phantom experiment demonstrated that the CT value and susceptibility value positively and negatively correlated with CaCO_3_ concentration, respectively (43). These studies above could support our result of the negative correlation between magnetic susceptibility and CT values in cortical bone.

This work has several limitations. First, the sample size was relatively small, which could lead to statistical bias in correlation analysis. Second, this study only conducted experiments on young and healthy subjects, making the study cohort fairly homogeneous in age and healthy conditions. The feasibility of UTE-QSM on patients with OA or osteoporosis should be further involved to explore the potential applications of UTE-QSM for clinical purposes. Third, a higher isotropic image resolution could be better for visualizing the cortical bone and articular cartilage. Fourth, although the registration performances between two continuous UTE scans and between CT and UTE images were both visually checked and were reasonable, future studies should use quantitative metrics to evaluate the registration accuracy.

In conclusion, we demonstrated the feasibility of UTE-QSM for simultaneous susceptibility mapping of cortical bone and articular cartilage *in vivo*. UTE-QSM may serve as a valuable imaging tool for investigating the knee joint.

## Data Availability Statement

The original contributions presented in the study are included in the article/**Supplementary Material**. Further inquiries can be directed to the corresponding author.

## Ethics Statement

The studies involving human participants were reviewed and approved by the Human Ethics Committee of Shanghai Jiao Tong University. The patients/participants provided their written informed consent to participate in this study.

## Author Contributions

MZ: methodology, data acquisition, data analysis, interpretation of findings, and manuscript drafting. ZL, HQW, and TC: methodology and data acquisition. YL, FY, and YZ: conceptualization, methodology, supervision, and manuscript editing. HJW: conceptualization, methodology, supervision, interpretation of findings, funding acquisition, and manuscript editing. All authors contributed to the article and approved the submitted version.

## Funding

This study was supported by the National Natural Science Foundation of China (91949120).

## Conflict of Interest

The authors declare that the research was conducted in the absence of any commercial or financial relationships that could be construed as a potential conflict of interest.

## Publisher’s Note

All claims expressed in this article are solely those of the authors and do not necessarily represent those of their affiliated organizations, or those of the publisher, the editors and the reviewers. Any product that may be evaluated in this article, or claim that may be made by its manufacturer, is not guaranteed or endorsed by the publisher.
